# Milling and Differential Sieving to Impact Buckwheat (*Fagopyrum esculentum*) Flour Techno-Functional Properties and Steamed Buckwheat Cake Quality

**DOI:** 10.3390/foods15091501

**Published:** 2026-04-25

**Authors:** Cailin Niu, Sevenur Sarıkaya, Meiling Ren, Junhong Feng, Fayin Ye

**Affiliations:** 1 College of Food Science, Southwest University, Chongqing 400715, China; 13156355992@163.com (C.N.); sevenur.98@gmail.com (S.S.); rmling_4020@163.com (M.R.); 18275689456@163.com (J.F.); 2 Chongqing Key Laboratory of Speciality Food Co-Built by Sichuan and Chongqing, Chongqing 400715, China; 3 Chongqing Engineering Research Center for Sweet Potato, Chongqing 400715, China

**Keywords:** buckwheat flour, sieving, particle size, steamed cake, buckwheat starch

## Abstract

Variations in the particle size of cereal flour could influence its techno-functional properties and affect the quality of the end products. In this study, common buckwheat (*Fagopyrum esculentum*) seeds were milled and then sieved into five fractions (≥200, 150–200, 100–150, 80–100, and 60–80 mesh). Proximate analysis showed that the protein and ash contents of buckwheat flour decreased with decreased particle size, whereas the starch content increased. Reducing the particle size did not change the A-type crystalline structure and the short-range ordered structure of buckwheat starch, whereas the buckwheat batter flowability, foaming properties and foam stability of the batter supernatant increased. The steamed buckwheat cakes made from ≥100-mesh flour showed a desirable appearance, cross-sectional structure, color, flavor, and texture. Pearson correlation analysis revealed that the starch content and relative crystallinity of buckwheat flour were significantly positively correlated with its pasting parameters and the textural properties (springiness, cohesiveness, resilience) and overall acceptability of steamed buckwheat cake, whereas the protein, lipid, and *β*-sheet content of buckwheat flour showed the opposite trend. This study demonstrated that differential sieving caused a difference in particle size and chemical composition, which were key variables governing the processing performance of buckwheat flour and important to the quality of its end products.

## 1. Introduction

Common buckwheat (*Fagopyrum esculentum* Moench) is an annual Polygonaceae crop grown globally for its nutrition and health benefits. In 2024, global cultivation covered 5.08 million hectares, yielding 5.05 million tons ([1]). Russia, China, Ukraine, and the USA are the top producers. Buckwheat seeds weigh 27.1–37.2 g per thousand and are rich in starch (up to 70 g/100 g), protein (10–15 g/100 g), fiber (10–12 g/100 g), fatty acids, vitamins, flavonoids, and minerals [2]. It is used in culinary dishes worldwide and processed into flour or groats for foods like Japanese soba, Korean naengmyeon, French galettes, Russian blini, Chinese fagao, wantuo, and helao, as well as bread, pancakes, and muffins in North America [3].

Steamed cake, a traditional staple or snack in China and other countries, is known for its fluffy texture, mild sweetness, and cultural significance. It has regional names such as fagao (China), jeungpyeon (Korea), idli (India), and bánh men (Vietnam). Its preparation is started either from naturally fermented cereal flour (e.g., rice, millet, oats) mixed with water to form a batter, or from cereal/pseudocereal (e.g., buckwheat, quinoa) flours blended with water, sugar, yeast, and microbial starters to form a batter. The batter is fermented at 25–37 °C for 40–60 min, and then steamed at 100 °C for several minutes to obtain the steamed cake. Unlike gluten-containing Chinese steamed bread, steamed cake is gluten-free; its aerated structure forms from gas bubbles, which are trapped during microbial fermentation and stabilized by starch retrogradation during cooling [4]. The quality of steamed cakes hinges on the properties of the fermented batter, a particle–liquid semi-fluid system [4].

Previous studies highlight the critical role of flour particle size in product quality. Cheng et al. [5] identified *D*_50_ = 65.86 µm as optimal for extruded buckwheat noodles. Kim & Shin [6] found that reducing rice flour particle size from 180 µm to 75 µm, the hardness, springiness, and the air cell size of gluten-free rice cupcakes decreased, while rice flour with particle size < 125 µm was suitable for producing gluten-free rice cupcakes with higher overall acceptability. Jima, Abera, & Kuyu [7] found that teff flour particles < 180 µm improve flatbread (injera) quality. Similarly, Xu et al. [8] reduced buckwheat flour particle size from 100 to 10 μm by airflow ultrafine grinding and improved its hydration properties, facilitating its broader application in foods.

Flour with varying particle sizes can be obtained through sieving or grinding without sieving. Sieving yields flours with the desired size and distribution but alters chemical composition across fractions, whereas grinding without sieving maintains chemical uniformity but may cause starch damage and protein denaturation, impairing final product quality [9]. Despite this, no studies have examined the effect of flour particle size on steamed buckwheat cake quality. Wang et al. [10] found that buckwheat hulls at cell-scale (50 μm) improved steamed rice cake quality when incorporated into rice flour, suggesting potential for dietary fiber enrichment. We hypothesized that reducing buckwheat flour particle size by differential sieving would enhance soluble component release and starch granule liberation, thereby affecting steamed buckwheat cake quality. To test this, buckwheat grain was ground and sieved into five fractions (≥200, 150–200, 100–150, 80–100, and 60–80 mesh) using sequential sieving with mesh sizes of 200, 150, 100, 80, and 60. The mechanistic effects of particle size on raw materials, processing behavior, and the final product were systematically investigated. Meanwhile, the chemical composition, structural characteristics, and physicochemical properties of the flours and the quality of the resulting steamed buckwheat cakes were analyzed. This study offers a scientific basis for optimizing cereal flour processing and expanding gluten-free product development potential.

## 2. Materials and Methods

### 2.1. Materials

Dehulled buckwheat (food-grade) was purchased from Harbin Shidakang Grain Processing Co., Ltd. (Heilongjiang, China). Table sugar was sourced from Chongqing Lanbo Food Co., Ltd. (Chongqing, China), and active dry yeast was from Angel Yeast Co., Ltd. (Hubei, China). The chemicals and solvents used in this study were of analytical grade.

### 2.2. Preparation of Buckwheat Flour

In this experiment, 8 kg of hulled buckwheat was ground into flour in 10 batches of 800 g each using a grinder (ZX-2500Y, Taihe Industry and Trade Co., Ltd., Wenzhou, Zhejiang, China), for 1 min per batch. The ground samples were combined and sieved sequentially through steel sieves (60–200 mesh). Starting with the 200-mesh sieve, the pass-through fraction was collected as BWF200. The remaining material was then sieved through decreasing mesh sizes (150, 100, 80, and 60), with the pass-through fractions labeled as BWF150, BWF100, BWF80, and BWF60, respectively.

### 2.3. Determination of the Chemical Composition of Buckwheat Flour

The moisture content of buckwheat flour was determined according to ISO 712-1:2024, (Cereals and cereal products—Determination of moisture content—Part 1: Reference method, International Organization for Standardization (ISO): Geneva, Switzerland, 2024). Protein content was measured using the Kjeldahl method according to ISO 20483:2013, (Cereals and pulses—Determination of the nitrogen content and calculation of the crude protein content—Kjeldahl method, International Organization for Standardization (ISO): Geneva, Switzerland, 2013). Lipid content was determined according to GB 5009.6-2025 (National Food Safety Standard—Determination of Fat in Foods: Method 1: Soxhlet Extraction, Beijing, China, 2025), and ash content was measured based on GB 5009.4-2016 (National Food Safety Standard—Determination of Ash in Foods, Beijing, China, 2016). Carbohydrate content was calculated by difference, and starch content was determined using an enzymatic method with a starch assay kit (GOD) [11]. The water activity of buckwheat flour was determined using a water activity meter (Wuxi Huake Instrument & Meter Co., Ltd., Wuxi, China).

### 2.4. Determination of the Particle Size Distribution of Buckwheat Flour

The particle size of buckwheat flour was measured using a laser particle size analyzer (Mastersizer 3000, Malvern Panalytical Ltd., Worcestershire, UK). Prior to the test, the instrument was cleaned at 2000 rpm. Buckwheat flour was dispersed in deionized water in a beaker and stirred at 2400 rpm until the obscuration reached 8–20%. The parameters were set as follows: starch refractive index value, 1.59; test speed, 2400 rpm. The D_10_, D_50_, D_90_, D[4,3], and D[3,2] were recorded.

### 2.5. Structural Characterization of Buckwheat Starch and Its Proteins

The structural characteristics of starch and its proteins in buckwheat flour and fermented buckwheat batter were measured via X-ray diffraction (XRD) and Fourier transform infrared spectroscopy (FTIR). Before the measurements, the fermented buckwheat batter was freeze-dried (SCIENTZ-10ND, Xinzhi Freeze Drying Equipment Co., Ltd., Hangzhou, Zhejiang, China), ground with a grinder (SHM2005, Zhejiang Supor Furniture Products Co., Ltd., Hangzhou, Zhejiang, China), and passed through a 100-mesh sieve for analysis. All samples were placed in a desiccator, and the moisture was equilibrated for 24 h.

The crystalline structure of buckwheat starch from the buckwheat flour and the freeze-dried fermented counterpart was analyzed using an X-ray powder diffractometer (X’Pert^3^ Powder, PANalytical B.V., Almelo, the Netherlands) operating at 40 kV and 40 mA with Cu-K*α* radiation (*λ* = 1.54 nm). The measurement conditions were based on the method reported by Li et al. [12], with modifications to accommodate the experimental samples: diffraction angle (2*θ*) scanning ranged from 4° to 40°, the scanning rate was 2°/min, and the step size was 0.02°. The relative crystallinity (RC, %) was calculated using MDI-Jade 6 software (Materials Data Inc., Livermore, CA, USA) by determining the *A*_c_ and *A_a_* and applying Equation (1):

(1) $$RC=\frac{A_{c}}{A_{c}+A_{a}}\times 100\%$$

where *A_c_* represents the total area of all crystalline diffraction peaks, and *A_a_* represents the total area of all amorphous diffraction peaks.

The FTIR spectra of buckwheat flour and freeze-dried fermented flour were recorded using an FTIR spectrometer equipped with an ATR accessory with a ZnSe crystal (Spectrum 100, PerkinElmer Instruments Co., Ltd., Waltham, MA, USA) over the wavenumber range of 4000–600 cm^−1^ with a resolution of 4 cm^−1^ and 32 scans per sample. The bands at 995, 1022, and 1047 cm^−1^ were used to characterize the short-range order structure of starch. The Omnic 8.0 software (Thermo Fisher Scientific, Madison, WI, USA) was used to deconvolute the FTIR bands at 1200–800 cm^−1^ and to calculate the absorbance ratios (*R*_1047/1022_ and *R*_1022/995_). The proportions of protein secondary structures (*α*-helix, *β*-sheet, *β*-turn, and random coil) were analyzed in the amide I region (1600–1700 cm^−1^) using PeakFit 4.2 software (SeaSolve Software Inc., San Jose, CA, USA) [13].

### 2.6. Rapid Viscosity Analyzer (RVA)

The pasting properties of buckwheat flour were measured using a Rapid Visco Analyzer (RVA-TecMaster, Perten Scientific Instruments Ltd., Stockholm, Sweden). A 3.0 g sample (dry basis) and 25 g deionized water were mixed and transferred into an RVA canister. The test was set at the speed of 960 r/min and held for 10 s at 50 °C in the beginning, slowed down to 160 r/min for 1 min at 50 °C, heated to 95 °C at a rate of 12 °C/min for 2.5 min, then cooled to 50 °C and maintained for 2 min [14]. The pasting parameters, including peak viscosity (PV, cP), trough viscosity (TV, cP), final viscosity (FV, cP), breakdown (BD, cP), and setback (SB, cP), were recorded.

### 2.7. Preparation of Yeast-Fermented Buckwheat Batter

Buckwheat batter was prepared by mixing 50 g of buckwheat flour (varying particle sizes), 7.5 g sugar, 50 mL deionized water, and 0.5 g active dry yeast [10]. The batter was fermented for 1 h at 35 °C and 70% relative humidity in a constant-temperature and -humidity incubator (BSC-150, Boxun Medical Biological Instrument Co., Ltd., Shanghai, China).

### 2.8. Rheological Measurements

The apparent viscosity and shear stress of the buckwheat batter before and after fermentation were tested using a rheometer (DHR-2, TA Instruments, New Castle, DE, USA). The measurement procedure was adapted from the method reported by Chen et al. [15], with modifications. The sample was placed between two parallel plates (40 mm diameter, 1 mm gap) of a rheometer. The steady-shear measurements were conducted at 0.5% strain and a shear rate (*γ*) between 0.01 s^−1^ and 100 s^−1^. The experiment was conducted at 25 °C. The variation in apparent viscosity (Pa·s) and shear stress (Pa) with increasing shear rate (s^−1^) was recorded.

### 2.9. Interfacial Measurements

The buckwheat batter before and after fermentation (obtained as described in Section 2.6) was transferred into 50 mL centrifuge tubes and centrifuged at 10,000 rpm for 20 min using a high-speed centrifuge (L535-1, Hunan Xiangyi Laboratory Instrument Development Co., Ltd., Changsha, Hunan, China). The resulting supernatant was separated from the pellet and used for subsequent analysis.

#### 2.9.1. Determination of Surface Tension

The surface tension (mN/m) of the supernatant obtained from buckwheat batter before and after fermentation at the air–water interface was measured using an automatic surface tensiometer equipped with a Wilhelmy plate (19 × 10 × 0.1 mm) (Sigma 700, Boerlin Technology Co., Ltd., Gothenburg, Sweden). The measurement procedure was adapted from the method reported by Qian et al. [16], with modifications. Briefly, 20 mL of the supernatant was transferred into a 25 mL glass cup, and the aluminum plate was centrally positioned and immersed 20 mm into the supernatant throughout the measurement. The total measurement time was 30 min. Before and after each measurement, the plate was cleaned with ethanol and sterilized using an alcohol flame.

#### 2.9.2. Determination of Foaming Properties

The foaming properties of the supernatant extracted from fermented buckwheat batter were measured using a foaming analyzer (DFA100, KRÜSS Scientific Instruments Co., Ltd., Hamburg, Germany). Briefly, 50 mL of the supernatant was transferred into a cylindrical glass tube using a syringe. The measurement conditions were as follows: stirring time, 15 s; rotation speed, 7000 rpm; and total measurement time, 20 min. At the end of the test, the total foam height (H_foam_, mm), the number of bubbles produced per square millimeter (BC, μm^2^), and the average bubble area (MBA, mm^−2^) were recorded.

### 2.10. Preparation of Steamed Buckwheat Cake

The fermented buckwheat batter (from Section 2.3) was portioned into 40 g aliquots and transferred into cake molds (7 cm × 4.5 cm × 3.3 cm). The batter was steamed at 100 °C for 25 min using a steamer (Yonghui Superstores Co., Ltd., Fuzhou, Fujian, China) and then cooled to room temperature for further analysis.

### 2.11. Characterizing Physical Properties of Steamed Buckwheat Cake

The specific volume of steamed buckwheat cakes was measured by the rapeseed displacement method [14], calculated as the volume-to-weight ratio (mL/g). The samples were cut into a uniform cylindrical shape (diameter 24 mm, height 12 mm) for testing texture properties. Textural properties were assessed using a texture analyzer (TA.XT Plus, Stable Micro System, Godalming, Surrey, UK) with a P36/R probe and a 50 kg load cell, based on the method of Pang et al. [17] with modifications: strain, 50%; pre-test speed, 3 mm/s; post-test speed, 1 mm/s; test speed, 3 mm/s; delay between the two compressions, 5 s; and trigger force, 5 g. Each sample underwent a minimum of ten replicates, with hardness (g), springiness, adhesiveness (g·s), cohesiveness, chewiness (g), and resilience recorded.

### 2.12. Sensory Evaluation of Steamed Buckwheat Cake

Before the formal evaluation, 20 panelists (12 females and 8 males, 21–30 years of age) from the College of Food Science, Southwest University (Chongqing, China) were trained in the sensory attributes, definitions, and scoring criteria used in this study, and then participated in the sensory evaluation. The sensory evaluation method was adapted from Wang et al. [18], with appropriate modifications. The panel assessed five parameters: color (20), aroma (20), appearance (20), texture (20), and overall acceptability (20). Each attribute was evaluated using a 20-point hedonic scale (as detailed in Table A1). All samples used for sensory evaluation were prepared with uniform dimensions (24 mm in diameter and 12 mm in height), sequentially numbered, and placed on five separate plates. The panelists were not informed of the sample formulations or flour information. Each panelist evaluated each sample once. Evaluations were conducted in a clean and bright food laboratory at 20–25 °C, with purified water provided to panelists to minimize sample flavor interference.

### 2.13. Statistical Analysis

All measurements were performed in three independent biological replicates, with data presented as mean ± standard deviation. Graphs were plotted using Origin 2025 (OriginLab Corporation, Northampton, MA, USA). One-way analysis of variance (ANOVA) was conducted via SPSS 26.0 (IBM, Armonk, NY, USA), and differences among samples were assessed by Duncan’s multiple range test (*p* < 0.05). Correlation analysis was performed using Pearson’s correlation coefficient, which was visualized as a heatmap using Origin 2025 (OriginLab Corporation, Northampton, MA, USA). Asterisks indicated the significance levels of the correlations: *, *p* ≤ 0.05; **, *p* ≤ 0.01; ***, *p* ≤ 0.001.

## 3. Results and Discussion

### 3.1. Chemical Composition and Particle Size Distribution Analysis

Table 1 summarizes the chemical compositions of buckwheat flour with varying particle sizes, assessed by differential sieving. Fine flours (BWF100, BWF150, and BWF200) exhibited higher moisture content than coarse flours (BWF60 and BWF80), indicating uneven moisture distribution and varying hydration capacities among grain components. Protein and carbohydrate contents ranged from 22.74 g/100 g to 5.29 g/100 g and 69.38 g/100 g to 90.52 g/100 g, respectively, likely due to milling and sieving effects. The proteins were mostly concentrated in the aleurone layer and embryo [3], while carbohydrates were distributed in the endosperm; these tissues were ruptured to different extents upon milling. By sieving, proteins were enriched in coarse flours, while carbohydrates (mainly starch) were accumulated in fine flours. Notably, the BWF150 starch content was 1.82 times that of BWF60. The endosperm (70–80% starch) fragmented into fine particles during milling [19], concentrating starch in fine flour. Lipid content varied irregularly, peaking in BWF80, attributed to higher seed coat inclusion in large-particle flour and the oil-rich embryo’s proximity to the bran region. Lipids are primarily in the germ, with minimal presence (<0.4%) in the endosperm [19]. The ash content of BWF60 was 3.89 times that of BWF200. This was mainly because ash content decreased with sieving, as it is concentrated in the pericarp, germ, and testa [3], and coarse flour contained more bran. Overall, particle size significantly influenced chemical composition, reflecting distinct structural and physicochemical properties across flour fractions.

D_10_, D_50_, and D_90_, indicate the particle diameter below which 10%, 50%, and 90% of the particle volume are found, respectively. D[4,3] represents the volume-weighted mean diameter, whereas D[3,2] represents the surface area-weighted mean diameter. Table 1 shows the particle size distribution of buckwheat flour results. From BWF60 to BWF200, the D_50_ decreased significantly by 84.12% (*p* < 0.05). The D[4,3] of BWF60 was 5.44 times that of BWF200, while the D[3,2] value was 6.66 times that of BWF200. These indicated that sieving through screens of different mesh sizes effectively reduced the particle size of buckwheat flour. Qin et al. [20] screened dry-milled and wet-milled rice flours through 80-, 100-, 150-, and 200-mesh sieves, and then blended them at certain proportions. The results showed that when the sieve mesh increased from 80 to 200, the D_50_ value decreased significantly by 90.26%.

### 3.2. Structure Characteristics of Buckwheat Flour and Fermented Batter

#### 3.2.1. XRD Patterns of Buckwheat Starch in the Flour and Fermented Batter

**Figure 1:**
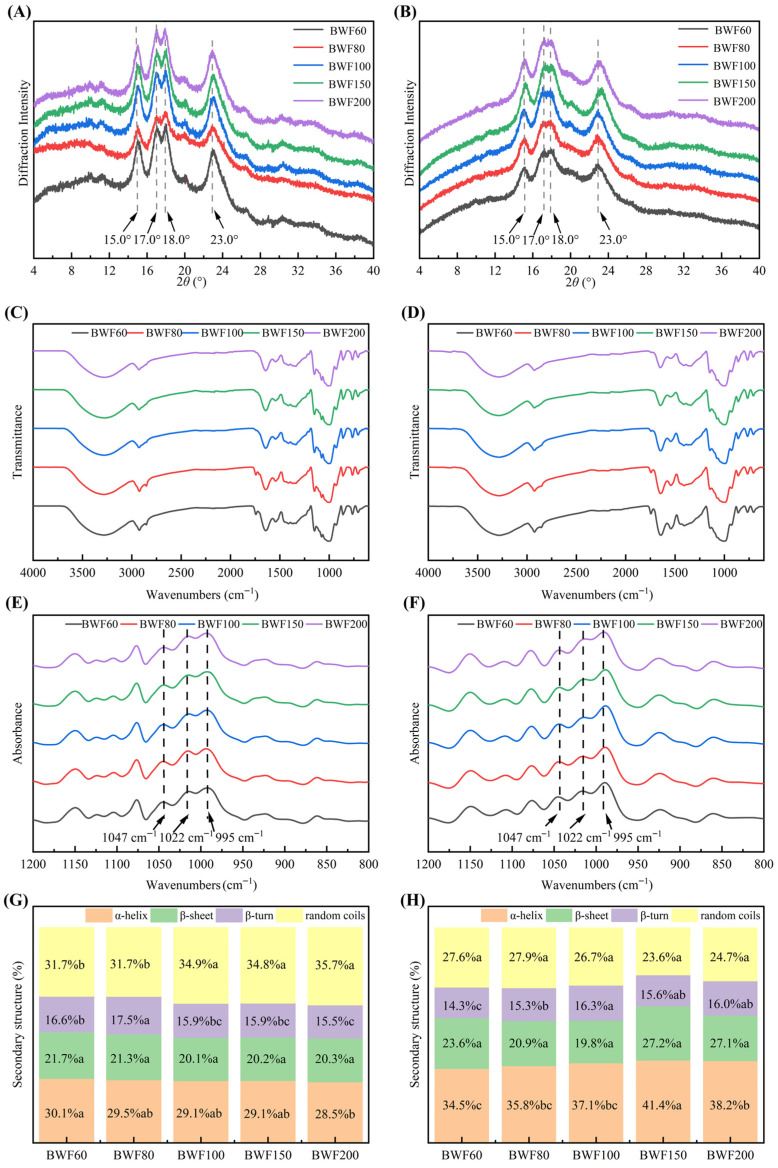
The X-ray diffraction patterns of unfermented (**A**) and freeze-dried fermented (**B**) buckwheat flour. FTIR spectra of unfermented (**C**) and freeze-dried fermented (**D**) buckwheat flour. Deconvolved spectra of unfermented (**E**) and freeze-dried fermented (**F**) buckwheat flour. Protein secondary structure of unfermented (**G**) and freeze-dried fermented (**H**) buckwheat flour. BWF200, BWF150, BWF100, BWF80, and BWF60 correspond to buckwheat flour fractions ≥200, 150–200, 100–150, 80–100, and 60–80 mesh using sequential sieving with progressively smaller mesh sizes (200, 150, 100, 80, and 60). Data are presented as the mean ± standard deviation from three independent replicates. Different lowercase letters in the same row indicate significant differences (*p* < 0.05).

Buckwheat flour is rich in starch, enabling X-ray diffraction (XRD) patterns to characterize the crystalline type of starch, while the calculated relative crystallinity reflects the long-range molecular order of starch in the sample. Figure 1A shows identical XRD patterns across particle sizes, with characteristic peaks at 15°, 17°, 18°, and 23° (2*θ*), confirming an A-type starch structure. Gao et al. [21] also reported that common whole buckwheat flour exhibited a typical A-type crystalline structure. This indicated that changing the particle size of buckwheat flour did not alter the crystalline structure of the starch. Fermented samples (Figure 1B) retained peak positions but exhibited reduced intensity, indicating decreased crystallinity [12].

Table 2 reveals significant crystallinity differences (30.18–32.40%) among particle sizes (*p* < 0.05), likely due to amylose content variation [15]. Milling promoted the liberation of starch granules and disrupted starch spatial structure, probably increasing damaged starch in BWF200. Compared with the relative crystallinity of the initial flour, that of fermented samples dropped to 13.48–16.34%, showing a decrease of >55%. It suggested that fermentation disrupted the crystalline structure of starch, possibly by breaking the double-helix structures [22].

#### 3.2.2. Short-Range Order of Buckwheat Starch in the Flour and Fermented Batter

Figure 1C shows the FTIR spectra of buckwheat flours with varying particle sizes. The absorption bands at 3000–3500 cm^−1^ reflect hydrogen-bonded group stretching vibrations. Bands at 2925 cm^−1^ (asymmetric -CH_2_- stretching) and 2853 cm^−1^ (symmetric -CH_2_- stretching) mainly correspond to non-starch polysaccharides (e.g., cellulose), while 1740 cm^−1^ indicates carbonyl stretching, 1637 cm^−1^ relates to amide I band and O-H stretching, and 1543 cm^−1^ is associated with N-H bending and C-N stretching. The 2853 cm^−1^ band intensity increased with particle size increment, attributed to the higher non-starch polysaccharide content in BWF60 compared to BWF200, enhancing the -CH_2_- stretching vibration at this band. The 800–1200 cm^−1^ region, a starch fingerprint zone [23], shows similar band positions across samples. Figure 1D reveals no significant band position shifts in fermented samples, but the intensity of the 800–1200 cm^−1^ region changed after fermentation. This aligned with Li et al. [12], who observed no band position changes in starch from co-fermented buckwheat flour dough after 6–24 h. Figure 1E and Figure 1F presented the FTIR fingerprint region (800–1200 cm^−1^) spectra of buckwheat flour before and after fermentation, respectively. Bands at 995, 1022, and 1047 cm^−1^ were sensitive to starch structural changes, reflecting short-range order of 1047 cm^−1^ (C-O-C stretching, crystalline structure), 1022 cm^−1^ (C-O-H stretching, amorphous regions), and 995 cm^−1^ (hydrogen bonds in *α*-1,4 and *α*-1,6 glycosidic bonds) [20]. The spectra were deconvoluted and calculated to obtain the absorbance ratios *R*_1047/1022_ (representing the degree of short-range order) and *R*_1022/995_ (representing the degree of double helices).

As shown in Table 2, the *R*_1047/1022_ values of buckwheat starch across particle sizes ranged from 1.22 to 1.33 (*p* > 0.05), indicating sieving did not significantly alter its short-range ordered structure. However, *R*_1022/995_ decreased from 0.65 to 0.43 with finer particle sizes, suggesting reduced double-helix content in finer starch. After fermentation, *R*_1047/1022_ increased to 1.48–1.64, while *R*_1022/995_ dropped by 58.1% (from 0.26 to 0.47), indicating enhanced short-range order. Similarly, Wang et al. [4] observed increased *R*_1047/1022_ in wheat starch after 90–120 min fermentation (FWS-90 min, FWS-120 min vs. FWS-60 min), suggesting that fermentation strengthens intermolecular interactions and promotes starch molecule aggregation.

#### 3.2.3. Secondary Structure of Buckwheat Proteins in the Flour and Fermented Batter

The amide I region (1600–1700 cm^−1^), primarily arising from the C=O stretching vibrations of peptide bonds, was sensitive to conformational changes and thus used to evaluate protein secondary structure. Specifically, ordered elements include *β*-sheets (1610–1640 cm^−1^ and 1680–1690 cm^−1^) and *α*-helix (1650–1660 cm^−1^), while disordered elements comprise random coils (1640–1650 cm^−1^) and *β*-turns (1660–1680 cm^−1^ and 1690–1700 cm^−1^) [24]. As shown in Figure 1G, all particle size fractions consisted of nearly equal proportions of ordered and disordered elements. The *α*-helix content decreased from 30.1% to 28.5% as particle size decreased, with BWF200 showing a significant 5% reduction compared to BWF60 (*p* < 0.05). This decline was likely due to differences in buckwheat flour particle size, which lead to variations in protein content and types and aggregation degree among fractions with different particle sizes. Kamal et al. [25] fractionated seed proteins from common buckwheat, identifying 270 proteins in the embryo and 163 in the endosperm, with 33 proteins common to both tissues. In addition, the random coil content increased significantly by 12.6% (*p* < 0.05), while *β*-sheet content remained unchanged (*p* > 0.05). The effect of fermentation on protein secondary structure is shown in Figure 1H. After fermentation, the ratio of ordered structures (*α*-helix and *β*-sheet) to disordered counterparts (*β*-turn and random coil) shifted to approximately 6:4, indicating a fermentation-induced transformation from disordered to ordered conformations. The *α*-helix content increased by 43.3% compared to unfermented flour, suggesting a more ordered and stabilized protein conformation. This increase may be attributed to (1) protein hydrolysis generating short peptides and protein aggregation promoting hydrogen bond formation, both of which favor *α*-helix stability [26]; and (2) the potential contribution of newly synthesized microbial proteins during fermentation.

### 3.3. Physicochemical Properties of Buckwheat Batter

#### 3.3.1. Rheological Properties of Buckwheat Batter

**Figure 2:**
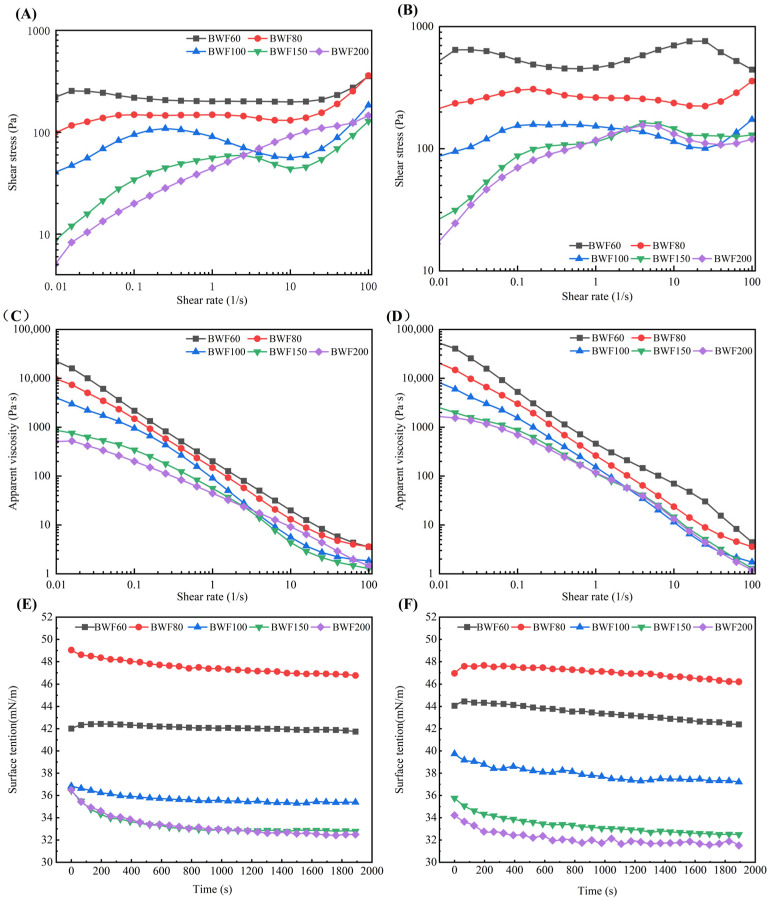
Shear stress vs shear rate of unfermented (**A**) and fermented (**B**) buckwheat batter. Apparent viscosity vs shear rate of unfermented (**C**) and fermented (**D**) buckwheat batter. Surface tension vs time of supernatant obtained from unfermented (**E**) and fermented (**F**) buckwheat batter. BWF200, BWF150, BWF100, BWF80, and BWF60 correspond to buckwheat flour fractions ≥200, 150–200, 100–150, 80–100, and 60–80 mesh using sequential sieving with progressively smaller mesh sizes (200, 150, 100, 80, and 60).

Rheology, describing material deformation and flow, is a key indicator for steamed cake processing, as batter rheological properties dictate gas retention and final product volume [27]. Figure 2A shows the shear stress behavior of unfermented batter. At 0.01 s^−1^, shear stress decreased with finer particle size, indicating higher zero-shear viscosity in coarser flours due to stronger particle interactions. In the low-shear region (0.01–0.12 s^−1^), all batters showed increasing stress with shear rate, exhibiting pseudoplastic flow as the internal network was disrupted. Between 0.12 and 10.00 s^−1^, shear stress decreased for BWF100 and BWF150 due to particle–aggregate breakdown and realignment, whereas it continued to rise for BWF200 owing to cluster formation under shear [27]. BWF60 and BWF80 showed little change, likely because their tightly packed particles resisted shear-induced rearrangement [28]. Above 10 s^−1^, stress increased for all samples. It suggested that the hydrodynamic forces dominated, promoting particle hydro-clustering [28]. Figure 2B showed the effect of fermentation on shear stress. Fermentation increased the shear stress (at 0.01 s^−1^) of all samples compared to the unfermented batter (Figure 2A), suggesting that it strengthened the batter structure. This was likely due to protein hydrolysis exposing more water-binding sites, thereby raising viscosity and flow resistance [29]. Figure 2C,D show the data of apparent viscosity vs. shear rate for unfermented and fermented buckwheat batters. All batters displayed shear thinning (non-Newtonian) behavior, with apparent viscosity decreasing as shear rate increased. This resulted from the alignment of soluble polysaccharides and proteins with the flow field and the disruption of inter-chain hydrogen bonds [27]. Notably, BWF150 and BWF200 showed a Newtonian plateau between 0.12 and 1.0 s^−1^, which might be attributed to flow regime disruption leading to hydro-cluster jamming [29]. In addition, fermented samples (Figure 2D) exhibited greater apparent viscosity than their unfermented counterparts. This might be attributed to microbial disruption of cell structures, releasing non-starch polysaccharides, and the entrapment of bubbles that hindered batter flow at low shear rates.

#### 3.3.2. Interfacial Properties of the Supernatant of Buckwheat Batter

Surface tension, governed by the migration of surface-active molecules to the air–water interface, reflects foam-stabilizing ability, and thus indicates the potential for stabilizing bubbles in the batter. In this context, the surface tension of the supernatant of unfermented and fermented batters was assayed. The samples in Figure 2E show decreasing surface tension over time, as proteins and lipids diffused to and adsorbed at the interface. The decrease was more rapid for BWF150 and BWF200 than for BWF60, BWF80, and BWF100, indicating a higher concentration of surface-active constituents in the former. This was likely due to (1) reduced particle size exposing more polar groups and water-binding sites [30]; and (2) lower batter viscosity (Figure 2C), facilitating the diffusion of surface-active constituents. Notably, the curves of BWF150 and BWF200 nearly overlapped, suggesting that beyond 150-mesh, particle size ceased to be a determining factor. As shown in Figure 2F, fermentation lowered the surface tension of supernatants from BWF80, BWF150, and BWF200, probably due to increased particle surface irregularity and partial disintegration, which released additional surface-active components [22]. After fermentation, the previously overlapping curves of BWF150 and BWF200 diverged, with BWF200 exhibiting lower tension, indicative of more pronounced fermentation in this sample. For BWF60 and BWF100, although the absolute tension values increased, the rate of tension reduction within the 0–1800 s measurement window was enhanced, implying that fermentation also induced more surface-active constituents in these batters [31]. Overall, the low surface tension and rapid tension reduction observed in supernatants of fine buckwheat flour batters were conducive to bubble stability and the formation of small, uniform gas cells in the batter, ultimately contributing to product quality [26].

**Figure 3:**
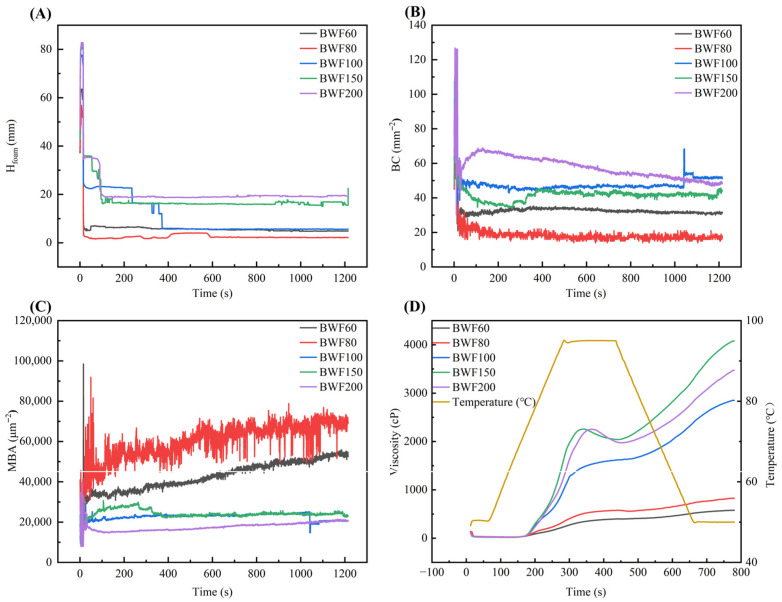
Foaming properties of supernatants obtained from fermented buckwheat batter. (**A**) Total foam height (H_foam_), (**B**) number of bubbles produced per square millimeter (BC), and (**C**) average bubble area (MBA). (**D**) Pasting properties of buckwheat flour with varying particle sizes. BWF200, BWF150, BWF100, BWF80, and BWF60 correspond to buckwheat flour fractions ≥200, 150–200, 100–150, 80–100, and 60–80 mesh using sequential sieving with progressively smaller mesh sizes (200, 150, 100, 80, and 60).

The gas-cell structure of steamed buckwheat cakes originates from bubbles trapped in the batter during steaming. To characterize this, the foaming properties of the fermented batter supernatant were assessed using a foam analyzer, recording foam height (H_foam_), bubble density (BC), and mean bubble area (MBA). Foamability refers to the continuous phase’s ability to entrap bubbles, while foam stability reflects its capacity to retain gas over time. As shown in Figure 3A, H_foam_ increased as buckwheat flour particle size decreased, indicating stronger foaming capacity in supernatants from fine freeze-dried fermented flours (BWF150 and BWF200). This aligned with their pronounced surface-tension reduction (Figure 2F), which lowered the energy required for foam generation [32]. BC represents the number of bubbles produced per unit area, indicating the foam density. Greater BC indicated a finer, more uniform aerated structure in the steamed buckwheat cakes. As shown in Figure 3B, BC was low for coarse-flour supernatants (BWF60, BWF80). For BWF200, BC initially rose, then decreased to levels similar to BWF100 and BWF150. Finer flours thus produced denser, more stable foam, as small bubbles packed tightly, enhancing resistance to deformation and drainage [33]. MBA represents the average bubble size within the foam, indicating bubble coalescence and liquid drainage over time. As shown in Figure 3C, MBA increased rapidly over time for BWF60 and BWF80 supernatants, but rose more slowly and remained lower for BWF100, BWF150, and BWF200. Smaller bubbles (as in fine-flour supernatants) were more stable due to lower surface energy, uniform interfacial tension, and tight packing, whereas larger bubbles were prone to rupture [32]. In summary, supernatants from coarser flour batters (BWF60, BWF80) formed unstable foam with bubbles that coalesced into larger sizes, while those from finer flour batters (BWF100, BWF150, and BWF200) exhibited better foam stability, maintaining small, stable bubbles that support a refined product microstructure.

### 3.4. Pasting Properties

During steaming, starch granules in the buckwheat batter gelatinized by absorbing water and swelling irreversibly, leading to amylose leaching and increased pasting viscosity. Subsequent rapid cooling induced starch short-term retrogradation, forming a gel network that trapped gas and ultimately determined the volume and texture of the cake. Given the central role of starch gelatinization in product structure formation, the pasting properties of buckwheat flours with different particle sizes were analyzed using a rapid viscosity analyzer (RVA). As shown in Figure 3D, all samples exhibited distinct rising viscosity as temperature increased from 50 °C to 95 °C, with BWF60 and BWF80 showing a slower increase rate than the others. During the 95 °C holding period, BWF60, BWF80, and BWF100 pastes maintained good thermal stability, whereas BWF150 and BWF200 showed a marked viscosity decrease. In the cooling phase, viscosity increased for all samples due to starch retrogradation. Overall, BWF60 and BWF80 exhibited restricted gelatinization, while BWF100, BWF150, and BWF200 displayed normal pasting behavior. This might be because BWF150 and BWF200 had smaller particle sizes and higher starch contents, allowing starch granules to have a higher degree of granule hydration and swelling. Although BWF100 had high starch content, its granule swelling might have been restricted and influenced by non-starch components.

The pasting parameters of buckwheat flour with different particle sizes are shown in Table 3. Peak viscosity (PV), indicating the maximum water absorption and swelling capacity of starch, was low for BWF60 and BWF80 due to tightly packed starch granules within intact endosperm cell walls, which limited gelatinization [30]. PV increased significantly as particle size decreased, with BWF100 being 3.08 times higher than BWF60 (*p* < 0.05), primarily due to extensive endosperm rupture and enhanced water absorption. No significant difference was observed between BWF150 and BWF200, likely because both contained abundant liberated starch with similar content. Trough viscosity (TV) reflected the structural stability of the swollen paste under heat and shear. It showed little decrease after PV for BWF60, BWF80, and BWF100, as starch remained largely enclosed within cell structures. In contrast, TV for BWF150 and BWF200 decreased significantly after PV, attributed to cell structure rupture, making their gelatinization behavior resemble purified starch [34]. Breakdown (BD = PV − TV) reflected the starch granule resistance at high temperature upon shearing. It increased significantly with finer particle size (*p* < 0.05). BD was lower for BWF150 than BWF200, suggesting better thermal stability for BWF150, likely because its starch remained somewhat packed, whereas BWF200 had more severely disrupted cells, reducing shear resistance [30]. Final viscosity (FV) represented the paste thickness after cooling and was related to the degree of amylose leaching and the presence of non-starch components in the paste. FV increased significantly with decreasing particle size (*p* < 0.05). FV of BWF150 was 7.06 times that of BWF60, due to greater amylose leaching, forming a stable network [34]. However, FV decreased for BWF200, possibly because soluble proteins hindered short-term retrogradation. Setback (SB = FV − TV) indicated the degree of short-term retrogradation. It increased with decreasing particle size, peaked at BWF150, then declined at BWF200. The FV/TV ratio, also characterizing short-term retrogradation, varied significantly but irregularly with particle size (*p* < 0.05). These trends suggested that particle size influences buckwheat starch short-term retrogradation, likely through differences in leached amylose and the inhibiting effects of non-starch components like proteins and non-starch polysaccharides.

### 3.5. Effect of Particle Size on the Quality Attributes of Steamed Buckwheat Cakes

#### 3.5.1. Appearance and Specific Volume of Steamed Buckwheat Cakes

**Figure 4:**
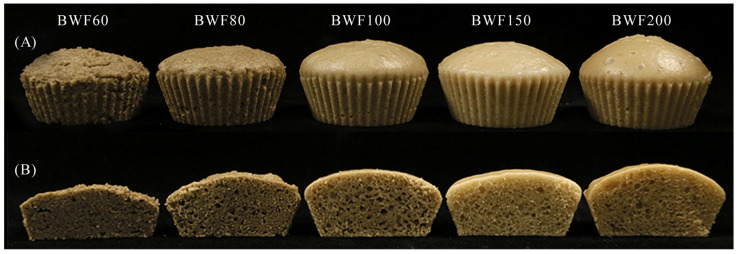
Photos of steamed buckwheat cakes prepared from buckwheat flour with varying particle sizes (**A**) and crumb structures (**B**). BWF200, BWF150, BWF100, BWF80, and BWF60 correspond to buckwheat flour fractions ≥200, 150–200, 100–150, 80–100, and 60–80 mesh using sequential sieving with progressively smaller mesh sizes (200, 150, 100, 80, and 60).

As shown in Figure 4, particle size markedly influenced the appearance and internal structure of steamed buckwheat cakes. Cakes made from coarse BWF60 flour were small in volume and had a shriveled surface (Figure 4A), likely due to the batter’s high shear stress and poor flowability (consistent with Figure 2), which restricted spreading in the mold. As particle size decreased, this shriveling was alleviated, with BWF100 producing a smooth surface, as a result of reduced viscosity and improved batter flowability. Cake volume increased with finer particle sizes, primarily because batters from fine flour exhibited stronger gas retention, allowing gas expansion during heating to form a fuller structure. Cross-sectional views (Figure 4B) revealed that BWF60 cakes had few pores, BWF80 and BWF100 cakes contained more large pores, while BWF150 and BWF200 cakes displayed dense, uniform pores. This pore structure improvement was likely attributed to the superior interfacial properties of the supernatant from fine-flour batters.

Specific volume, a key indicator of steamed cake quality, reflects the degree of volume expansion and gas retention capacity. As shown in Table 4, the flour particle size markedly influenced this parameter. Steamed cakes made from ≤100-mesh flours (BWF60, BWF80, and BWF100) showed similar specific volumes (1.33–1.50 mL/g, *p* > 0.05). In contrast, using ≥150-mesh flours (BWF150 or BWF200) significantly increased specific volume, with the 200-mesh sample reaching 2.08 mL/g. It showed a 38.67% increase over the 100-mesh counterpart. As shown in Section 3.3.2, BWF150 and BWF200 exhibited higher H_foam_ and BC, indicating that finer flour (≥150-mesh) enhanced both gas production during fermentation and the batter’s gas-holding capacity, leading to a more aerated structure. This aligns with Burešová et al. [35], who attributed higher loaf volume in buckwheat bread made from finer flour due to its elevated damaged starch content. In summary, particle size is a key determinant of the appearance and structural characteristics of steamed buckwheat cakes, with an optimal (moderate-to-fine) range contributing to enhanced product quality.

#### 3.5.2. Texture Properties of Steamed Buckwheat Cakes

The textural properties of steamed buckwheat cakes are presented in Table 4. Hardness was defined as the maximum force required to deform the food. The hardness of the cake significantly decreased with finer particle size (*p* < 0.05). Compared to cakes made from BWF60, the hardness of those from BWF80, BWF100, BWF150, and BWF200 was only 72.13%, 38.74%, 27.56%, and 22.30%, respectively. The steamed buckwheat cakes were starch gel food with a porous structure, which had hardness primarily governed by two factors: the starch gel matrix and the pore structure. Finer flours (BWF100, BWF150, and BWF200) had higher starch content (Table 1) and formed a more continuous gel network. Although short-term retrogradation (FV/TV from RVA, Figure 3D) showed irregular trends, the superior foaming properties and stability of batters made from finer flour resulted in more internal pores after steaming, effectively reducing hardness [10]. Springiness represented the ability of a sample to recover to its original height after the first compression. As particle size decreased, more amylose leaked out and participated in forming these structures, leading to a significant increase in springiness (*p* < 0.05). Kim & Shin [6] also reported that as the particle size of rice flour decreased from <180 μm to <125 μm, the springiness of rice cupcakes significantly increased from 0.75 to 0.83. Adhesiveness, the force required to separate the food from the probe after compression, is related to the cake surface properties and amylose leaching during steaming. Cakes from finer flours (BWF100, BWF150, and BWF200) showed lower adhesiveness than those from coarser ones (BWF60, BWF80). This was likely because finer flour released more amylose into the aqueous phase during steaming, forming a more complete gel via starch short-term retrogradation, thereby reducing surface stickiness. Cohesiveness, reflecting the sample’s deformation resistance, indicated the interaction among different ingredients [36]. RVA tests indicated an enhanced short-term retrogradation with decreasing particle size. Thus, a reduction in particle size led to a significant increase in cohesiveness (*p* < 0.05). Chewiness (calculated as hardness × springiness × cohesiveness) represents the energy required to chew food to a swallowable state. High hardness typically leads to high chewiness. As particle size decreased, chewiness significantly decreased (*p* < 0.05), which was consistent with the findings for wheat flour steamed bread [17]. Resilience characterizes the sample’s ability for rapid elastic recovery after compression. The resilience of steamed buckwheat cakes varied significantly with particle size (*p* < 0.05). BWF150 exhibited the maximum resilience, indicating that cakes made from this flour had the best elasticity and softness. In summary, reducing buckwheat flour particle size favored the production of steamed cakes with moderate hardness and desirable elasticity.

#### 3.5.3. Sensory y Properties of Steamed Buckwheat Cakes

Sensory evaluation, reflecting product acceptability and quality, assessed steamed buckwheat cakes made from flours of varying particle sizes based on appearance, color, aroma, texture, and overall acceptability. As demonstrated in Table 4, particle size significantly influenced these sensory attributes. Appearance scores improved with decreasing particle size for BWF60, BWF80, and BWF100 (*p* < 0.05), but showed no significant difference between BWF150 and BWF200. Color scores also increased significantly as particle size decreased; BWF60 and BWF80 scored lower than the finer flours (BWF100, BWF150, and BWF200). This aligns with Figure 4A, where cakes from coarser flours (BWF60, BWF80) appear darker, likely due to their higher polyphenol content [35]. Conversely, finer flours (BWF100, BWF150, and BWF200) were richer in starch (Table 1) and produced brighter cakes with higher color scores. Aroma scores increased with finer particle size, suggesting enhanced fermented flavor development. This was probably attributed to the larger specific surface area of fine flour, which improved microbial activity during fermentation. Texture scores significantly improved for BWF100, BWF150, and BWF200. Finer flours formed a more continuous gel network during steaming, correlating with the more uniform pore structure observed in Figure 4B. Overall acceptability scores were significantly lower for BWF60, BWF80, and BWF100 compared to BWF150 and BWF200 (*p* < 0.05). Conclusively, using buckwheat flour passed through 150- or 200-mesh sieves guaranteed the desired overall acceptability of steamed buckwheat cakes.

### 3.6. Correlation Analysis

**Figure 5:**
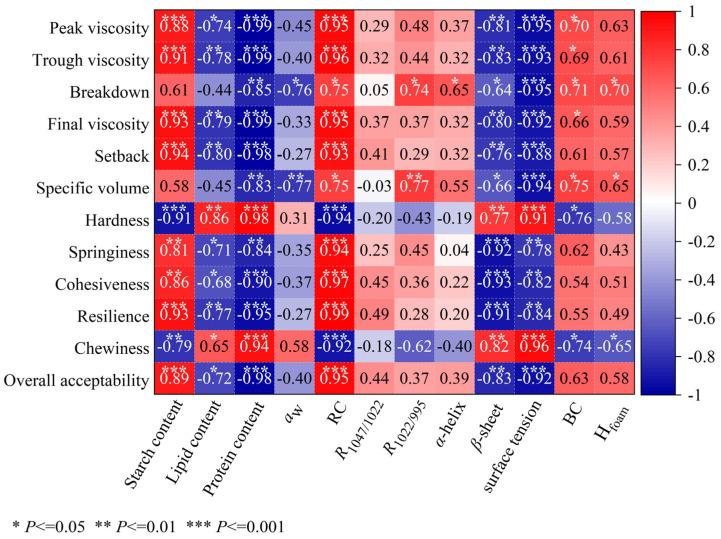
Pearson correlation analysis between chemical composition and physicochemical properties of buckwheat flour, the interfacial properties of the supernatant of fermented buckwheat batter, and quality parameters of steamed buckwheat cakes.

Pearson correlation analysis (Figure 5) revealed relationships between buckwheat flour compositional and physicochemical properties, interfacial properties, the results of the supernatant of fermented batters and steamed cake quality. Pasting properties were significantly correlated with chemical composition, i.e., peak, trough, final, and setback viscosities showed negative correlations with lipid and protein contents. The proteins and lipids in the buckwheat flours likely led to a restriction of starch gelatinization and retrogradation. Breakdown viscosity was significantly negatively correlated with *a*_w_ (*p* < 0.05), as higher *a*_w_ indicated more non-starch components, preventing swollen starch granule rupture during RVA testing. Pasting parameters were significantly positively correlated with RC, while breakdown viscosity was positively correlated with *R*_1022/995_ (*p* < 0.05), reflecting increased amorphous regions in starch. Breakdown was also significantly positively correlated with α-helix (*p* < 0.05), attributed to protein-type variations [25], and negatively correlated with *β*-sheet content (*p* < 0.05), suggesting that *β*-sheet-rich proteins hindered starch gelatinization. The pasting parameters (peak, trough, final, breakdown, and setback viscosities) were significantly negatively correlated with surface tension (*p* < 0.001). Peak, trough, final, and breakdown viscosities were significantly positively correlated with BC (*p* < 0.05). Breakdown viscosity was also significantly positively correlated with H_foam_ (*p* < 0.05).

The specific volume of steamed buckwheat cake showed a strong negative correlation with protein content (*p* < 0.01), indicating that higher protein levels impede volume expansion. It was also negatively correlated with *a*_w_ (*p* < 0.01), *β*-sheet content (*p* < 0.05), and surface tension (*p* < 0.001), likely because the flours with higher *a*_w_ and more *β*-sheet structures, or the higher surface tension of the supernatant obtained from fermented buckwheat batter, restricted gas retention during steaming. Conversely, specific volume was significantly positively correlated with *R*_1022/995_ (*p* < 0.01). Higher values of *R*_1022/995_, reflecting a greater proportion of amorphous starch regions, promoted starch gelatinization and the formation of a gas-retaining gel network. It was also significantly positively correlated with BC and H_foam_ (*p* < 0.05), indicating that as the supernatant obtained from fermented buckwheat batter formed small bubbles or exhibited better foam stability, the steamed cakes tended to have a larger specific volume.

The hardness and chewiness of steamed buckwheat cake showed strong negative correlations with starch content (*p* < 0.01), consistent with the observed texture results (Table 4), i.e., finer flour (higher starch) yielded softer, less chewy cakes. These parameters were also negatively correlated with RC (*p* < 0.001) and BC (*p* < 0.05), but positively correlated with *β*-sheet content and surface tension (*p* < 0.01), indicating that buckwheat proteins were richer in *β*-sheets, and the higher surface tension of the supernatant increased the hardness and chewiness. Springiness, cohesiveness, and resilience were negatively correlated with *β*-sheet, protein and lipid content, as well as surface tension, suggesting that lipids and proteins (particularly with higher *β*-sheet content) hindered the formation of a continuous gel network essential for cake texture. Conversely, these three textural attributes were significantly positively correlated with RC (*p* < 0.001). In addition, overall acceptability was extremely significantly positively correlated with starch content and RC (*p* < 0.001), and negatively correlated with lipid, protein and *β*-sheet content, as well as surface tension.

## 4. Conclusions

This study investigated the effects of particle size (≥200, 150–200, 100–150, 80–100, and 60–80 mesh) on the properties of buckwheat flour and the quality of steamed buckwheat cakes. As particle size decreased, flour composition shifted, i.e., moisture, carbohydrate, and starch contents increased, while protein, lipid, and ash content decreased. Finer flours (≥100-mesh) showed higher starch content, lower water activity, and greater starch relative crystallinity (RC), which declined after fermentation but remained higher than in coarser flours (<100-mesh). The short-range order of starch (*R*_1047/1022_) was unaffected by particle size, but the double-helix order (*R*_1022/995_) declined with particle size reduction. Protein secondary structure was less affected by particle size than by fermentation. Functionally, supernatants from ≥100-mesh batters had lower surface tension. Foam properties (H_foam_, BC, MBA) varied significantly, with ≥100-mesh batters exhibiting superior foam stability. Pasting viscosities were lower in <100-mesh flours due to intact cellular structures, but increased in ≥100-mesh flours with greater rupture of endosperm tissues (*p* < 0.05). In final product quality, finer flours (≥100-mesh) improved cake appearance, crumb structure, color, flavor, and texture, attributed to enhanced batter flowability, gas retention, and starch gelatinization. Correlation analysis revealed that starch content and RC positively correlated with pasting viscosities, springiness, and overall acceptability (*p* < 0.05), while protein, lipid, and *β*-sheet content showed negative correlations. The findings highlight the significant influence of particle size on the properties of buckwheat flour and cake quality. For optimal steamed buckwheat cakes, ≥150-mesh flour is recommended based on sensory evaluation. However, observed effects were attributable not only to changes in particle size caused by differential degrees of sieving, but also to the redistribution of the basic components of buckwheat flour resulting from the sieving process. Further research should explore varietal differences and milling and modification strategies to better utilize buckwheat flour with specific particle size fractions.

## Figures and Tables

**Table 1:** Chemical composition and particle size distributions of buckwheat flour with different particle sizes.

	BWF200	BWF150	BWF100	BWF80	BWF60
Chemical composition					
Moisture (g/100 g wb)	12.41 ± 1.19 ^a^	11.68 ± 0.05 ^ab^	11.84 ± 0.41 ^ab^	11.26 ± 0.10 ^bc^	10.75 ± 0.11 ^c^
Carbohydrate (g/100 g db)	83.29 ± 0.56 ^b^	87.50 ± 0.62 ^a^	82.86 ± 0.62 ^b^	63.33 ± 1.19 ^c^	63.31 ± 0.56 ^c^
Starch (g/100 g db)	42.71 ± 0.70 ^a^	47.00 ± 3.20 ^a^	45.88 ± 3.49 ^a^	27.85 ± 2.85 ^b^	25.71 ± 2.29 ^b^
Protein (g/100 g db)	8.20 ± 0.96 ^c^	5.29 ± 0.49 ^d^	10.82 ± 0.48 ^b^	22.53 ± 0.95 ^a^	22.74 ± 0.35 ^a^
Lipid (g/100 g db)	7.18 ± 0.27 ^c^	6.19 ± 0.27 ^d^	5.03 ± 0.24 ^e^	9.83 ± 0.32 ^a^	8.77 ± 0.47 ^b^
Ash (g/100 g db)	1.33 ± 0.02 ^c^	1.02 ± 0.05 ^d^	1.29 ± 0.03 ^c^	4.31 ± 0.06 ^b^	5.18 ± 0.09 ^a^
Particle size distributions					
D_10_ (μm)	10.06 ± 1.05 ^d^	51.70 ± 0.57 ^bc^	38.87 ± 7.52 ^cd^	80.75 ± 26.23 ^b^	222.33 ± 23.18 ^a^
D_50_ (μm)	56.10 ± 1.27 ^e^	99.05 ± 0.21 ^d^	124.00 ± 1.00 ^c^	253.50 ± 4.95 ^b^	353.33 ± 2.08 ^a^
D_90_ (μm)	113.00 ± 2.83 ^e^	161.00 ± 0.00 ^d^	223.00 ± 1.00 ^c^	414.50 ± 9.19 ^b^	510.33 ± 13.50 ^a^
D[4,3] (μm)	59.45 ± 1.63 ^e^	102.00 ± 0.00 ^d^	129.33 ± 0.58 ^c^	254.50 ± 2.12 ^b^	323.67 ± 9.29 ^a^
D[3,2] (μm)	26.20 ± 1.98 ^a^	58.25 ± 0.92 ^a^	58.43 ± 1.34 ^a^	70.85 ± 3.18 ^a^	174.43 ± 144.26 ^a^

Carbohydrate (g/100 g db) = 100 − (protein + lipid + ash). BWF200, BWF150, BWF100, BWF80, and BWF60 correspond to buckwheat flour fractions ≥200, 150–200, 100–150, 80–100, and 60–80 mesh using sequential sieving with progressively smaller mesh sizes (200, 150, 100, 80, and 60). Data are presented as the mean ± standard deviation from three independent replicates. Different lowercase letters in the same row indicate significant differences (*p* < 0.05).

**Table 2:** Physical properties of buckwheat flour with different particle sizes.

	BWF200	BWF150	BWF100	BWF80	BWF60
Buckwheat flour					
*a*_w_	0.60 ± 0.01 ^c^	0.63 ± 0.01 ^b^	0.65 ± 0.01 ^a^	0.64 ± 0.01 ^ab^	0.65 ± 0.02 ^a^
RC (%)	83.29 ± 0.56 ^b^	87.50 ± 0.62 ^a^	82.86 ± 0.62 ^b^	63.33 ± 1.19 ^c^	63.31 ± 0.56 ^c^
*R*_1047/1022_	42.71 ± 0.70 ^a^	47.00 ± 3.20 ^a^	45.88 ± 3.49 ^a^	27.85 ± 2.85 ^b^	25.71 ± 2.29 ^b^
*R*_1022/995_	8.20 ± 0.96 ^c^	5.29 ± 0.49 ^d^	10.82 ± 0.48 ^b^	22.53 ± 0.95 ^a^	22.74 ± 0.35 ^a^
Buckwheat flour (freeze-dried after fermentation)					
RC (%)	16.00 ± 0.37 ^a^	16.34 ± 0.16 ^a^	16.08 ± 0.12 ^a^	14.35 ± 0.19 ^b^	13.48 ± 0.28 ^c^
*R*_1047/1022_	1.55 ± 0.04 ^ab^	1.64 ± 0.07 ^a^	1.60 ± 0.09 ^ab^	1.59 ± 0.01 ^ab^	1.48 ± 0.03 ^b^
*R*_1022/995_	0.47 ± 0.09 ^a^	0.31 ± 0.00 ^b^	0.28 ± 0.07 ^b^	0.26 ± 0.14 ^b^	0.30 ± 0.00 ^b^

*a*_w_: water activity; RC: relative crystallinity; *R*_1047/1022_: the FTIR-measured absorbance ratio of 1047 cm^−1^ to 1022 cm^−1^; *R*_1022/995_: the FTIR-measured absorbance ratio of 1022 cm^−1^ to 995 cm^−1^; BWF200, BWF150, BWF100, BWF80, and BWF60: corresponding to buckwheat flour fractions ≥200, 150–200, 100–150, 80–100, and 60–80 mesh using sequential sieving with progressively smaller mesh sizes (200, 150, 100, 80, and 60). Data are presented as the mean ± standard deviation from three independent replicates. Different lowercase letters in the same row indicate significant differences (*p* < 0.05).

**Table 3:** Pasting properties of buckwheat flour with different particle sizes.

	BWF200	BWF150	BWF100	BWF80	BWF60
PV (cP)	0.60 ± 0.01 ^c^	0.63 ± 0.01 ^b^	0.65 ± 0.01 ^a^	0.64 ± 0.01 ^ab^	0.65 ± 0.02 ^a^
TV (cP)	83.29 ± 0.56 ^b^	87.50 ± 0.62 ^a^	82.86 ± 0.62 ^b^	63.33 ± 1.19 ^c^	63.31 ± 0.56 ^c^
FV (cP)	42.71 ± 0.70 ^a^	47.00 ± 3.20 ^a^	45.88 ± 3.49 ^a^	27.85 ± 2.85 ^b^	25.71 ± 2.29 ^b^
BD (cP)	8.20 ± 0.96 ^c^	5.29 ± 0.49 ^d^	10.82 ± 0.48 ^b^	22.53 ± 0.95 ^a^	22.74 ± 0.35 ^a^
SB (cP)	16.00 ± 0.37 ^a^	16.34 ± 0.16 ^a^	16.08 ± 0.12 ^a^	14.35 ± 0.19 ^b^	13.48 ± 0.28 ^c^

PV: peak viscosity, TV: trough viscosity, BD: breakdown viscosity, FV: final viscosity, SB: setback viscosity. BWF200, BWF150, BWF100, BWF80, and BWF60: corresponding to buckwheat flour fractions ≥200, 150–200, 100–150, 80–100, and 60–80 mesh using sequential sieving with progressively smaller mesh sizes (200, 150, 100, 80, and 60). Data are presented as the mean ± standard deviation from three independent replicates. Different lowercase letters in the same row indicate significant differences (*p* < 0.05).

**Table 4:** Physical properties of buckwheat steamed cakes made from buckwheat flour with different particle sizes.

	BWF200	BWF150	BWF100	BWF80	BWF60
Specific volume (mL/g)	2.08 ± 0.26 ^a^	1.75 ± 0.24 ^b^	1.50 ± 0.05 ^c^	1.35 ± 0.05 ^c^	1.33 ± 0.01 ^c^
Texture					
Hardness (g)	909.73 ± 15.83 ^e^	1124.33 ± 98.84 ^d^	1580.54 ± 13.60 ^c^	2943.07 ± 132.72 ^b^	4079.83 ± 157.78 ^a^
Springiness	0.94 ± 0.09 ^a^	0.90 ± 0.11 ^b^	0.87 ± 0.18 ^c^	0.83 ± 0.05 ^d^	0.77 ± 0.08 ^e^
Adhesiveness (g·s)	−38.3 ± 2.90 ^b^	−20.65 ± 1.87 ^a^	−27.63 ± 4.76 ^a^	−79.96 ± 4.74 ^c^	−183.90 ± 11.74 ^d^
Cohesiveness	0.75 ± 0.22 ^a^	0.73 ± 0.09 ^b^	0.69 ± 0.04 ^c^	0.60 ± 0.07 ^d^	0.47 ± 0.09 ^e^
Chewiness (g)	522.95 ± 11.38 ^e^	761.17 ± 65.06 ^d^	973.91 ± 50.00 ^c^	1339.23 ± 112.67 ^b^	1521.92 ± 21.01 ^a^
Resilience	0.40 ± 0.08 ^b^	0.42 ± 0.03 ^a^	0.37 ± 0.04 ^c^	0.26 ± 0.03 ^d^	0.16 ± 0.03 ^e^
Sensory analysis					
Appearance	16.65 ± 1.87 ^a^	17.80 ± 1.47 ^a^	13.60 ± 2.16 ^b^	10.10 ± 2.59 ^c^	7.65 ± 2.60 ^d^
Color	16.50 ± 1.36 ^b^	18.25 ± 1.12 ^a^	13.75 ± 1.45 ^c^	9.75 ± 1.83 ^d^	7.70 ± 2.15 ^e^
Aroma	15.30 ± 2.81 ^a^	15.00 ± 3.40 ^a^	14.15 ± 2.21 ^ab^	13.20 ± 2.46 ^ab^	12.45 ± 4.25 ^b^
Texture	16.15 ± 1.95 ^ab^	17.25 ± 1.92 ^a^	14.55 ± 2.76 ^b^	11.25 ± 3.34 ^c^	9.90 ± 3.31 ^c^
Overall acceptability	17.50 ± 1.70 ^a^	16.65 ± 1.50 ^a^	12.85 ± 2.54 ^b^	10.05 ± 2.84 ^c^	8.05 ± 3.91 ^d^

BWF200, BWF150, BWF100, BWF80, and BWF60 correspond to buckwheat flour fractions of ≥200, 150–200, 100–150, 80–100, and 60–80 mesh using sequential sieving with progressively smaller mesh sizes (200, 150, 100, 80, and 60). Data are presented as the mean ± standard deviation from three independent replicates. Different lowercase letters in the same row indicate significant differences (*p* < 0.05).

## Data Availability

The original contributions presented in this study are included in the article. Further inquiries can be directed to the corresponding author.

## Appendix A

**Table A1:** Sensory scoring items and scoring criteria for steamed buckwheat cakes.

Index	Quality Description	Score
Appearance	Smooth surface without high volume	16–20
	Slightly smooth surface, moderate volume	10–15
	Shriveled, small volume	0–9
Color	Brightness and appeal of the surface color	16–20
	Slightly dull and acceptable	10–15
	Dull color and unappealing	0–9
Aroma	Strong fermented aroma and slightly sweet	16–20
	Slight fermented aroma and acceptable	10–15
	Sour and unpleasant odor, unacceptable	0–9
Texture	Fine, dense, and uniform pore distribution, fluffy texture	16–20
	Slightly uniform with a few large pores, slightly fluffy	10–15
	Large and unevenly distributed pores	0–9
Overall acceptability	Soft and elastic, easy to chew, non-stick	16–20
	Moderately soft and elastic, slightly difficult to chew, and sticky	10–15
	Difficult to chew, without elasticity, and very sticky	0–9

## References

[1] Food and Agriculture Organization of the United Nations (FAO) (2024). FAOSTAT Statistical Database [Data set]. https://www.fao.org/faostat/.

[2] Lin R., Sang L., Zhu Y., Wang Y., Liu X., Zhao L., Shen Q., Xue Y., Zhao Q. (2025). Buckwheat 2.0: From Climate-Resilient Crop to Personalized Nutrition Product—A Model Approach for Future Food Industry. Trends Food Sci. Technol..

[3] Cai Y.Z., Corke H., Wang D., Wrigley C.W., Corke H., Seetharaman K., Faubion J. (2016). Buckwheat: Overview. Encyclopedia of Food Grains.

[4] Wang H., Liu J., Zhang Y., Li S., Liu X., Zhang Y., Zhao X., Shen H., Xie F., Xu K. (2024). Insights into the hierarchical structure and physicochemical properties of starch isolated from fermented dough. Int. J. Biol. Macromol..

[5] Cheng J., Lei S., Gao L., Zhang Y., Cheng W., Wang Z., Tang X. (2022). Effects of Jet Milling on the Physicochemical Properties of Buckwheat Flour and the Quality Characteristics of Extruded Whole Buckwheat Noodles. Foods.

[6] Kim J.-M., Shin M. (2014). Effects of Particle Size Distributions of Rice Flour on the Quality of Gluten-Free Rice Cupcakes. LWT-Food Sci. Technol..

[7] Jima B.R., Abera A.A., Kuyu C.G. (2025). Effect of Particle Size on Compositional, Functional, Pasting, and Rheological Properties of Teff [*Eragrostis teff* (Zucc.) Trotter] Flour. Appl. Food Res..

[8] Xu Q., Zheng F., Cao X., Yang P., Xing Y., Zhang P., Liu H., Zhou G., Liu X., Bi X. (2021). Effects of Airflow Ultrafine-Grinding on the Physicochemical Characteristics of Tartary Buckwheat Powder. Molecules.

[9] Tian X., Sun B., Wang X., Ma S., Li L., Qian X. (2020). Effects of Milling Methods on Rheological Properties of Fermented and Non-Fermented Dough. Grain Oil Sci. Technol..

[10] Wang L., Guo Z., Wang H., Zou L., Li Z., Fang B., Qiu J. (2024). Effect of Buckwheat Hulls at Cell-Scale on Physiochemical and Textural Properties of Steamed Rice Cake. LWT.

[11] Gu Y., Qian X., Sun B., Ma F., Zhang H., Zhang Y., Wang L. (2022). Nutritional composition and physicochemical properties of oat flour sieving fractions with different particle size. LWT.

[12] Li X., Wei S., Gao Z., Zhao R., Wang Z., Fan Y., Cui L., Wang Y. (2023). The Influence of Cooperative Fermentation on the Structure, Crystallinity, and Rheological Properties of Buckwheat Starch. Curr. Res. Food Sci..

[13] Cao H., Wang X., Zhang Y., Song H., Liu C., Huang K., Lu J., Grimi N., Guan X. (2025). Enhancing the Texture and Modulating Digestive Behavior of Gluten-Free Quinoa Sponge Cakes via Microwave-Assisted Alkaline Amino Acid Treatment. Food Chem..

[14] Feng W., Zhang H., Wang R., Liu J., Zhao S., Chen L., Li Y. (2021). Modifying the internal structures of steamed rice cakes by emulsifiers for promoted textural and sensory properties. Food Chem..

[15] Chen N., Wang Q., Wang M.-X., Li N., Briones A.V., Cassani L., Prieto M.A., Carandang M.B., Liu C., Gu C.-M. (2022). Characterization of the Physicochemical, Thermal and Rheological Properties of Cashew Kernel Starch. Food Chem. X.

[16] Qian X., Sun B., Ma S., Liu C., Wang X. (2024). The Role of Lipids in Determining the Gas Cell Structure of Gluten-Free Steamed Oat Cake. Food Hydrocoll..

[17] Pang J., Guan E., Yang Y., Li M., Bian K. (2021). Effects of Wheat Flour Particle Size on Flour Physicochemical Properties and Steamed Bread Quality. Food Sci. Nutr..

[18] Wang Z., Yuan Z., Dou X., Yang W., Zhang H., Zhang Y., Chen F., Hao Y. (2025). Enhancement of Anti-Staling Properties of Rice Bread Through Fermentation Rice Flour with Three Lactic Acid Bacteria. Foods.

[19] Skřivan P., Chrpová D., Klitschová B., Švec I., Sluková M. (2023). Buckwheat Flour (*Fagopyrum esculentum* Moench)—A Contemporary View on the Problems of Its Production for Human Nutrition. Foods.

[20] Qin W., Lin Z., Wang A., Chen Z., He Y., Wang L., Liu L., Wang F., Tong L.-T. (2021). Influence of Particle Size on the Properties of Rice Flour and Quality of Gluten-Free Rice Bread. LWT.

[21] Gao L., Cheng W., Fu M., Wu D., Tang X. (2022). Effect of Improved Extrusion Cooking Technology Modified Buckwheat Flour on Whole Buckwheat Dough and Noodle Quality. Food Struct..

[22] Song M.-K., Guo X.-N., Zhu K.-X. (2025). Elucidating the Gas Cell Stabilization Mechanism of Buckwheat-Wheat Steamed Bread Induced by Transglutaminase: A Focus on the Foaming and Air-Water Interfacial Properties of Dough Liquor. Food Hydrocoll..

[23] Zhang L., Apea-Bah F.B., Chen X., Hornung P.S., Malunga L.N., Beta T. (2023). The Physicochemical and Structural Properties and in Vitro Digestibility of Pea Starch Isolated from Flour Ground by Milling and Air Classification. Food Chem..

[24] Zhang Y., Yin Y., Wu Y., Wang Z., Liu J., Guan X. (2025). GABA Enrichment Pathways in Oat and Buckwheat Stimulated by Combined Germination and *Lactiplantibacillus plantarum* Fermentation Treatment. Food Biosci..

[25] Kamal A.H.M., Jang I.-D., Kim D.-E., Suzuki T., Chung K.-Y., Choi J.-S., Lee M.-S., Park C.-H., Park S.-U., Lee S.H. (2011). Proteomics Analysis of Embryo and Endosperm from Mature Common Buckwheat Seeds. J. Plant Biol..

[26] Diowksz A., Sadowska A. (2021). Impact of Sourdough and Transglutaminase on Gluten-Free Buckwheat Bread Quality. Food Biosci..

[27] Liu Y., Yu S., Jiang P., Fu B., Qi L., Shang S. (2024). Effect of Exogenous Protein Substitution in Glutinous Rice Cake: Batter Rheology, Structure, and Retrogradation Behavior. J. Cereal Sci..

[28] Crawford N.C., Popp L.B., Johns K.E., Caire L.M., Peterson B.N., Liberatore M.W. (2013). Shear Thickening of Corn Starch Suspensions: Does Concentration Matter?. J. Colloid Interface Sci..

[29] Yang Q., Lyu Y., Wu Z., Li X., Liu K. (2024). Effect of Sourdough–Yeast Co-Fermentation on Physicochemical Properties of Corn Fagao Batter. Foods.

[30] Chen Z., Huang Q., Xia Q., Zha B., Sun J., Xu B., Shi Y.C. (2020). Intact Endosperm Cells in Buckwheat Flour Limit Starch Gelatinization and Digestibility in Vitro. Food Chem..

[31] Liu Y., Sun X.-H., Zhu K.-X., Guo X.-N. (2025). Effect of *Jiuniang* Pretreatment on the Quality of Tartary Buckwheat Dough and Steamed Bread: A Focus on the Protein of Dough and Interface Properties of Dough Liquor. J. Cereal Sci..

[32] Wang Z., Li S., Xu Z., Aryana S.A., Cai J. (2025). Advances and Challenges in Foam Stability: Applications, Mechanisms, and Future Directions. Capillarity.

[33] Song M.-K., Guo X.-N., Zhu K.-X. (2024). Alkali-Induced Protein Structural, Foaming, and Air–Water Interfacial Property Changes and Quantitative Proteomic Analysis of Buckwheat Sourdough Liquor. J. Agric. Food Chem..

[34] Huang Y., Sun X., Guo H., He X., Jiang J., Zhang G., Li W. (2021). Changes in the Thermal, Pasting, Morphological and Structural Characteristic of Common Buckwheat Starch after Ultrafine Milling. Int. J. Food Sci. Technol..

[35] Burešová I., Lullien-Pellerin V., Červenka L., Mlček J., Šebestíková R., Masaříková L. (2023). The Comparison of the Effect of Flour Particle Size and Content of Damaged Starch on Rice and Buckwheat Slurry, Dough, and Bread Characteristics. Foods.

[36] Roman L., Reguilon M.P., Gomez M., Martinez M.M. (2020). Intermediate Length Amylose Increases the Crumb Hardness of Rice Flour Gluten-Free Breads. Food Hydrocoll..

